# LINC01134: a pivotal oncogene with promising predictive maker and therapeutic target in hepatocellular carcinoma

**DOI:** 10.3389/fonc.2024.1265762

**Published:** 2024-02-21

**Authors:** Yutian Yu, Jialing Wang, Qingfa Guo, Hongliang Luo

**Affiliations:** ^1^ Department of Spleen and Stomach Diseases, Jiujiang Hospital of Traditional Chinese Medicine, Jiujiang, Jiangxi, China; ^2^ Department of Gastrointestinal Surgery, The Second Affiliated Hospital, Jiangxi Medical College, Nanchang University, Nanchang, Jiangxi, China; ^3^ Second Clinical Medical College, Nanchang University, Nanchang, Jiangxi, China

**Keywords:** hepatocellular carcinoma, LINC01134, tumorigenesis, tumor biomarker, therapeutic target

## Abstract

Hepatocellular carcinoma (HCC) represents a leading and fatal malignancy within the gastrointestinal tract. Recent advancements highlight the pivotal role of long non-coding RNAs (lncRNAs) in diverse biological pathways and pathologies, particularly in tumorigenesis. LINC01134, a particular lncRNA, has attracted considerable attention due to its oncogenic potential in hepatoma. Current research underscores LINC01134’s potential in augmenting the onset and progression of HCC, with notable implications in drug resistance. This review comprehensively explores the molecular functions and regulatory mechanisms of LINC01134 in HCC, offering a fresh perspective for therapeutic interventions. By delving into LINC01134’s multifaceted roles, we aim to foster novel strategies in HCC management.

## 1 Introduction

Liver cancer, predominantly hepatocellular carcinoma (HCC), is a gastrointestinal malignancy characterized by its insidious onset, rapid progression, restricted therapeutic efficacy, and dire prognosis. In 2020, liver cancer ranked as the sixth most prevalent cancer globally, with an incidence surpassing 900,000 new diagnoses (1). It also stood as the third leading cause of cancer-related mortality, accounting for over 830,000 deaths annually (1). Remarkably, China bore nearly half of the global liver cancer mortality burden (2, 3). Although the past decade has seen progress in liver cancer therapeutics (4), the long-term prognosis remains bleak, as evidenced by the low 5-year survival rate of 12.1% in China (5), and similarly unsatisfactory rates in affluent nations such as the United States of America (17.4%) (6). Therefore, unraveling the molecular intricacies of HCC and identifying new molecular markers and therapeutic avenues are of paramount importance.

Long noncoding RNAs (lncRNAs) are transcripts defined by their length—typically over 200 nucleotides—and limited protein-coding potential (7, 8). The biogenesis of lncRNAs is complex; they are transcribed by RNA Polymerase II, sharing certain characteristics with mRNAs, yet they also exhibit unique features such as less evolutionary conservation, fewer exons, and lower expression levels (9–11). Some lncRNAs are inefficiently spliced and terminate transcription independently of polyadenylation signals, resulting in nuclear retention and degradation mediated by the RNA exosome (12). Factors including transcriptional regulation, processing, and specific sequence motifs that recruit nuclear factors influence their nuclear localization. U1 small nuclear ribonucleoprotein (snRNP) binding sites and repeat elements play roles in lncRNA localization (13). A significant fraction of lncRNAs is exported to the cytosol via the Nuclear RNA export factor 1 (NXF1) pathway (14), where they undergo sorting processes, potentially associating with ribosomes for translation or localizing to mitochondria or exosomes, broadening their functional diversity. In essence, lncRNA biogenesis involves transcription, splicing, and sequence motifs, governing their nuclear retention and cytoplasmic export (15).

LncRNAs are classified based on their genomic location (intergenic, intronic, sense, and antisense) and size (small, medium, large) (16). Pioneering research has unveiled their crucial roles in various physiological processes, modulating gene expression at the epigenetic (17, 18), transcriptional (19–21), and post-transcriptional levels (20–22). LncRNAs interact with DNA, RNA, and proteins, influencing chromatin structure, gene transcription, RNA splicing, stability, and translation (15). They can serve as scaffolds for protein interactions, guide chromatin modifiers to specific genomic loci, or compete with microRNAs to regulate mRNA expression. Exploring the complexities of lncRNAs is poised to uncover new dimensions of gene regulation and cellular function.

Emerging data highlight the significant role of lncRNAs in human pathologies (23–25), especially in malignant tumors (26–29). Dysregulation of lncRNAs has been closely linked to tumor growth, metastasis, and resistance to chemotherapy (30–34), radiotherapy (35–38), and immune responses (39–41), underscoring their crucial involvement in cancer development.

**Figure 1:**
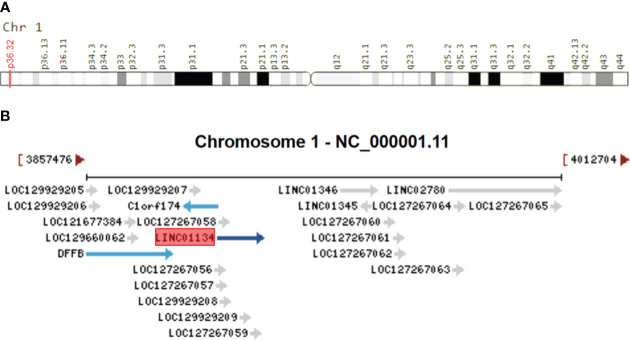
Genomic Overview of LINC01134. **(A)** Genomic location of LINC01134 sourced from the GeneCaRNA database (https://www.genecards.org/cgi-bin/carddisp.pl?gene=LINC01134), and **(B)** the genomic context depicting neighboring genes of LINC01134, derived from the NCBI database (https://www.ncbi.nlm.nih.gov/gene/100133612).

Homo sapiens long intergenic non-protein-coding RNA 1134 (LINC01134), also known as TLNC1, is a lncRNA gene located on Chromosome 1p36.32 (**Figure 1A**). This gene, consisting of four exons and spanning a sequence length of 15,044 nucleotides, plays a pivotal role in various biological processes (42–44). To provide context to the significance of LINC01134, it’s essential to consider its neighboring genes, which include C1orf174, LINC01346, LINC02780, and DFFB, as illustrated in **Figure 1B**. Understanding the genomic context of LINC01134 sheds light on its potential functional importance and its role in gene regulation. Recently, LINC01134 has gained attention for its role in the progression of HCC (42, 45). and it has been identified as a prognostic indicator for HCC patients (42–44). In addition, LINC01134 has been linked to an unfavorable prognosis in ovarian cancer (46), and signatures related to LINC01134 have shown superior predictive value for prognosis in low-grade glioma (47) and gastric cancer (48). Altered expression of LINC01134 has been observed in HCC tissues and cell lines, implicating it in the progression of HCC (42–45, 49–51).

To compile pertinent literature for a comprehensive analysis of LINC01134’s involvement in cancer biology and its prospective clinical relevance, we conducted a thorough systematic search across multiple databases. These databases included PubMed, Web of Science, ScienceDirect, Embase, SpringerLink, and Google Scholar, as well as Chinese databases such as China National Knowledge Infrastructure (CNKI), Weipu Information (VIP), and Wanfang Database. Our search utilized the keywords ‘LINC01134’ and ‘TLNC1’. We considered peer-reviewed articles published in English or Chinese up to October 1, 2023. The inclusion criteria were predefined, focusing on original studies that explored LINC01134’s expression, clinicopathological associations, and biological functions in human tumors.

In this review, we provide a comprehensive analysis, addressing aberrant expression patterns, molecular mechanisms, and the clinical significance of LINC01134 in HCC. This review aims to establish a theoretical foundation for potential future clinical applications, predict the functions and regulatory mechanisms of LINC01134, and delineate both the current research gaps and future directions.

## 2 LINC01134 and HCC

**Figure 2:**
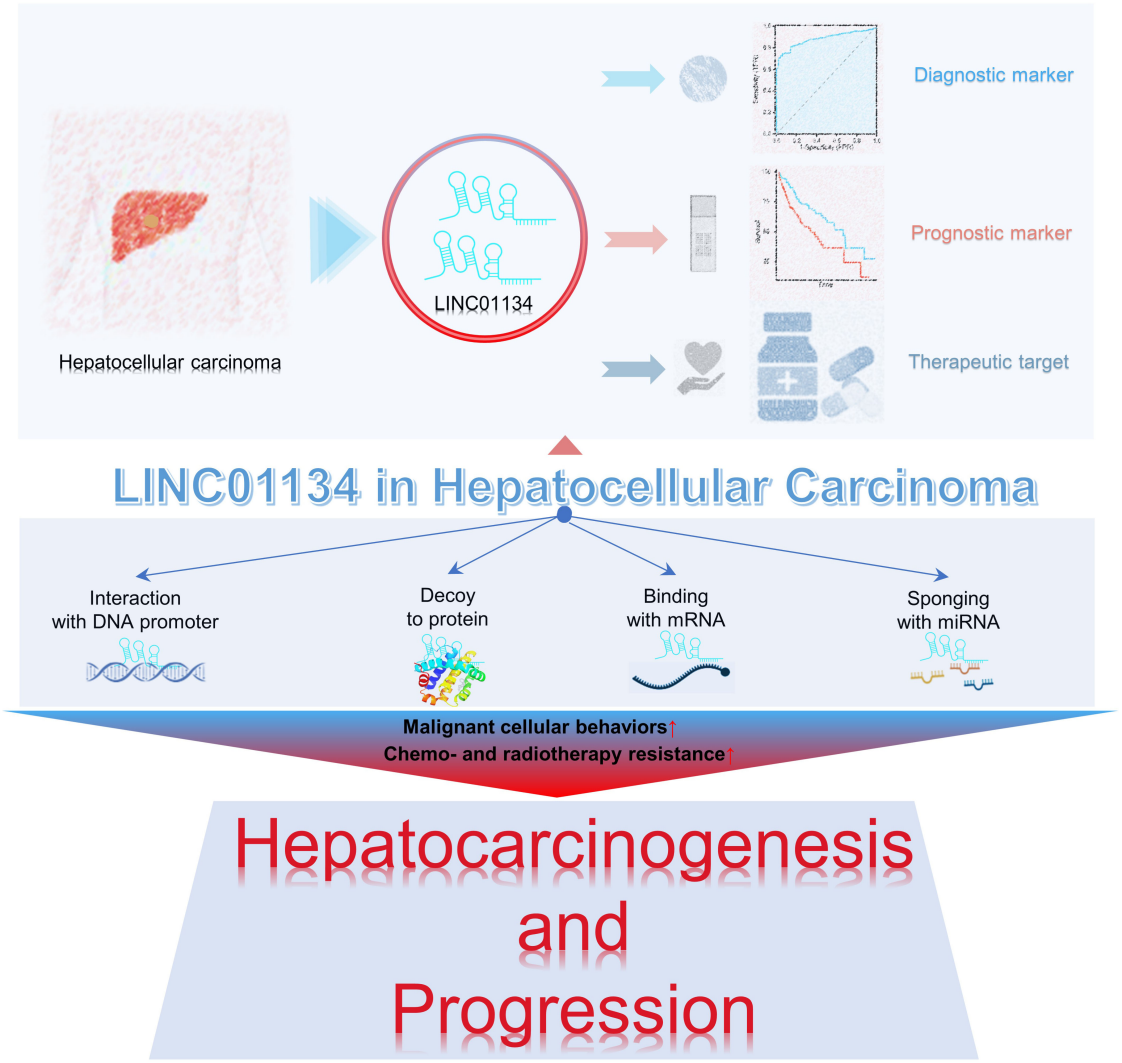
LINC01134 plays a role in the progression of HCC and demonstrates potential clinical significance as both a tumor marker and therapeutic target.

Research underscores the pivotal roles of LINC01134 in hepatocarcinogenesis and progression. LINC01134 functions through diverse mechanisms, including interactions with DNA promoters, decoy proteins, mRNA binding, and acting as a miRNA sponge, regulating malignant cellular behaviors, and contributing to tumor therapy resistance (**Figure 2**). It has also been identified as a promising prognostic and diagnostic tumor marker, along with its potential as a therapeutic target in hepatoma (**Figure 2**). In the following sections, we reviewed the multifaceted role of LINC01134 in HCC progression, metastasis, and its impact on chemo- and radiotherapy resistance. In addition, we examine the clinical applications of LINC01134 in HCC, focusing on prognosis, diagnosis, and therapy.

### 2.1 LINC01134 expression in HCC and its involvement in tumorigenesis

Understanding the expression patterns and functional role of LINC01134 in HCC provides insights into its potential as a valuable biomarker and therapeutic target. Recent experimental studies (**Table 1**) have provided compelling evidence regarding the significant upregulation of LINC01134 in HCC tissues and HCC cancer cell lines. Consistent associations have been observed between increased expression of LINC01134 and unfavorable clinical outcomes in HCC patients. These findings highlight the potential significance of LINC01134 in HCC, suggesting its viability as a biomarker or therapeutic target for this malignancy.

**Table 1:** LINC01134 expression, prognostic value, and related regulatory roles in hepatocellular carcinoma.

Author	Expression	Prognosis	Experiments	Functions	Mechanism	Signaling	Ref.
Rong et al.	Upregulated	OS	*In vitro*; *In vivo*	Cell cycle arrest, viability, proliferation, apoptosis, migration, invasion, and EMT; Tumor growth and metastasis	LINC01134/miR-324-5p/IGF2BP1/YY1 feedback loop	–	(42)
Yuan et al.	Upregulated	OS, DFS	*In vitro*; *In vivo*	Cell motility, proliferation, migration and invasion; Tumor growth and metastasis	LINC01134/TPR/p53 axis	p53 signaling	(45)
Wang et al.	Upregulated	OS, DFS	*In vitro*; *In vivo*	Cell migration, invasion; Tumor metastasis	LINC01134/ AKT1S1/p65 axis	NF-κB signaling	(43)
Zheng et al.	Upregulated	OS	*In vitro*	Cell proliferation	miR-4784/SSRP1 axis	–	(44)
Ma et al.	Upregulated	OS, PFS, DFS	*In vitro*; *In vivo*	Oxaliplatin resistance	LSD1/LINC01134/SP1/p62 axis	p62- mediated anti- oxidative stress response pathway	(49)
Wang et al.	Upregulated	–	*In vitro*; *In vivo*	Radio-resistance	LINC01134/miR-342-3p/MAPK1 axis	MAPK signaling	(50)
Kang et al.	Upregulated	OS, RFS	*In vitro*	Oxaliplatin resistance	LINC01134/Nrf2/GPX4 axis	GPX4 pathway	(51)
Li et al.	Upregulated	OS	*In vitro*	T cell migration	–	–	(52)

OS, Overall Survival; DFS, Disease-Free Survival; PFS, Progression-Free Survival; RFS, Recurrence-Free Survival; EMT, Epithelial-Mesenchymal Transition; IGF2BP1, Insulin-Like Growth Factor 2 mRNA Binding Protein 1; YY1, Yin-Yang 1; TPR, Translocated Promoter Region, Nuclear Basket Protein; AKT1S1, AKT Serine/Threonine Kinase 1 Substrate 1; SSRP1, Structure-Specific Recognition Protein 1; SP1, Specificity Protein 1; MAPK1, Mitogen-Activated Protein Kinase 1; Nrf2, Nuclear factor erythroid 2-related factor 2; GPX4, Glutathione Peroxidase 4; “-”: Indicates missing or not applicable data.

Moreover, elevated LINC01134 expression has been associated with various facets of HCC progression, including enhanced cell proliferation, migration, invasion, and tumor growth and metastasis (**Table 1**). It has also been implicated in processes such as epithelial-mesenchymal transition (EMT) (42), immune cell infiltration (specifically T cell migration) (52), and resistance to chemotherapy and radiotherapy (49–51) in HCC (**Table 1**). Furthermore, LINC01134 plays a regulatory role in HCC development by influencing key signaling pathways, including the Nuclear Factor Kappa B (NF-κB) inflammatory pathway (43), the MAPK signaling cascade (50), and the tumor suppressor p53 pathway (45), as well as genes involved in tumorigenesis and disease progression (**Table 1**). These insights are instrumental in comprehending the essential role of LINC01134 in the functional landscape of HCC.

### 2.2 The role of LINC01134 in HCC growth and metastasis

**Figure 3:**
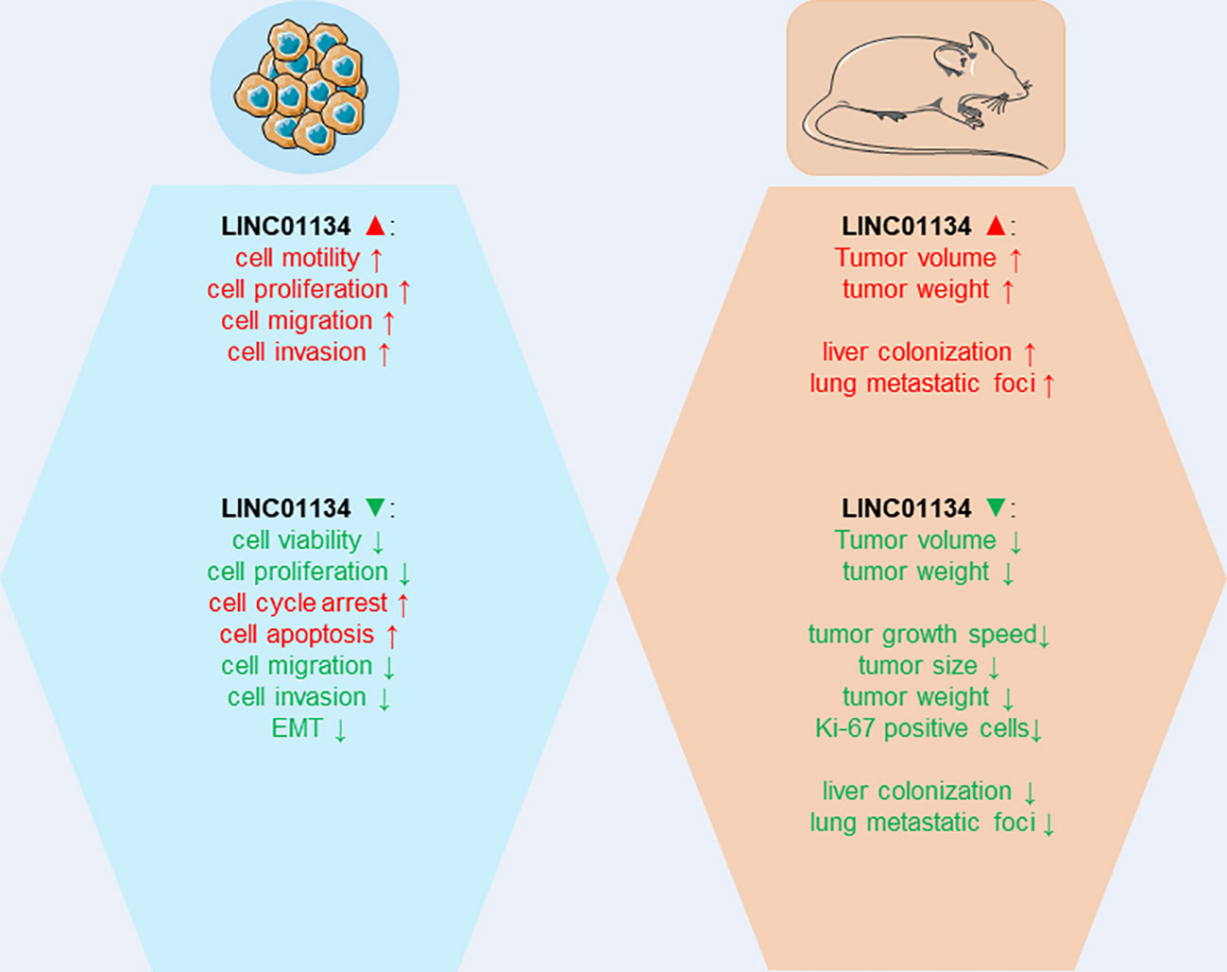
The impact of LINC01134 overexpression and silencing in cellular assays and mouse models.

LINC01134 has risen to prominence as a critical regulator of numerous tumor-associated cellular processes in HCC. The roles played by LINC01134 in modulating malignant biological behaviors, both *in vitro* and *in vivo*, are summarized in **Figure 3**. A series of studies (42–45) have demonstrated that the knockdown of LINC01134 results in decreased HCC cell viability, proliferation, migration, and invasion, and it inhibits the EMT process. Moreover, it induces cell cycle arrest and promotes cell apoptosis *in vitro*. LINC01134 silencing also inhibits tumor growth and metastasis *in vivo*. In contrast, ectopic expression of LINC01134 stimulates HCC cell viability, proliferation, migration, and invasion in HCC cell lines. Furthermore, overexpression of LINC01134 facilitates tumor growth and metastasis in mouse models.

**Figure 4:**
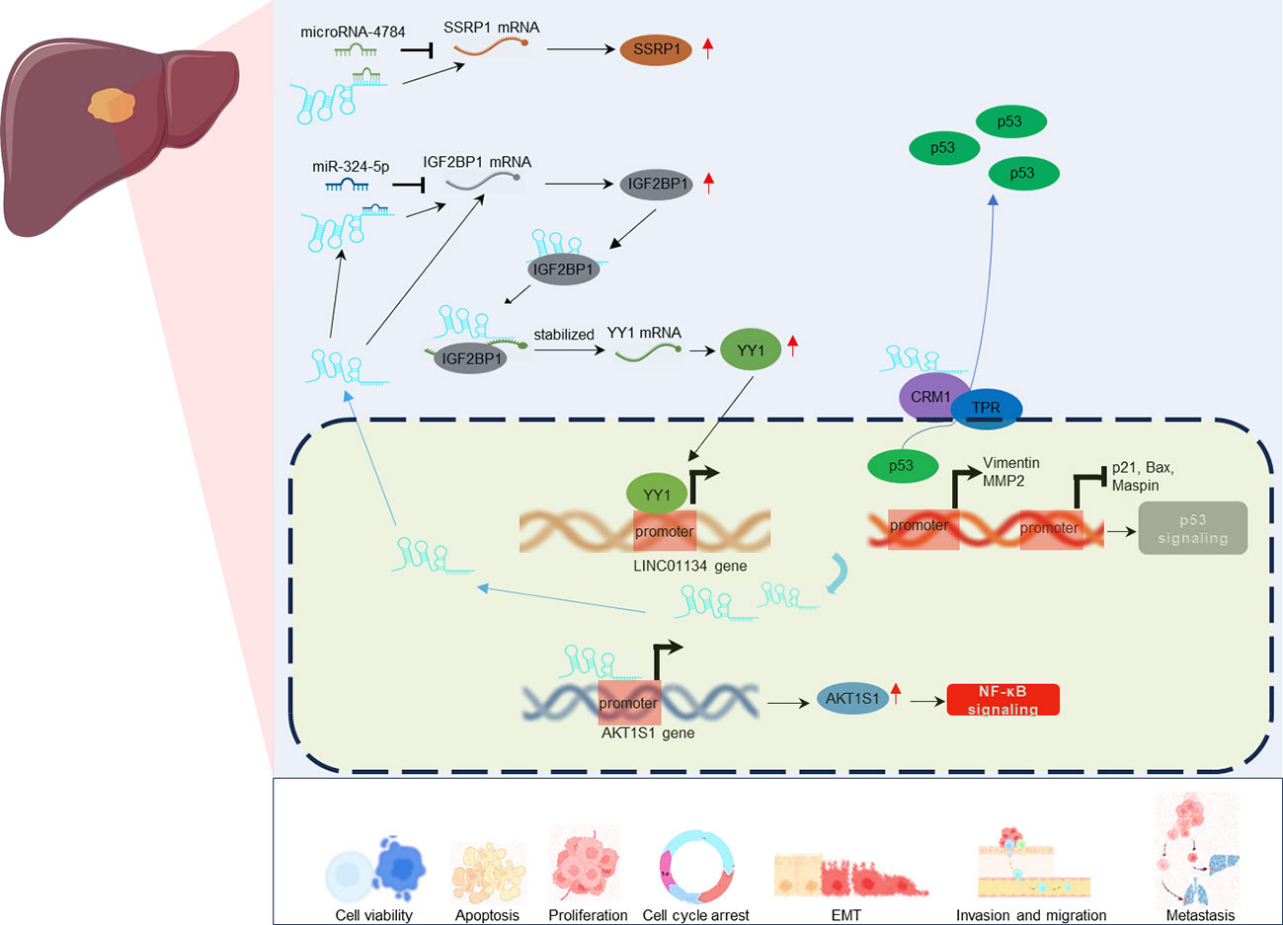
Regulatory mechanisms of LINC01134 in various tumor-associated cellular processes in HCC.

Mechanistically (**Figure 4**), LINC01134 plays a multifaceted role, functioning as an endogenous miRNA sponge, aiding transcriptional activation through interactions with DNA promoters, and modulating protein transport between the nucleus and cytoplasm. These mechanisms contribute to the diverse functional roles of LINC01134 in cellular processes. For instance, Rong et al. (42) demonstrated that LINC01134 acts as a sponge for miR-324-5p and interacts with Insulin-like growth factor 2 mRNA-binding protein 1 (IGF2BP1), thereby increasing the stability of Yin-Yang 1 (YY1) mRNA expression. YY1, a well-established transcription factor, plays a pivotal role in transcriptional regulation (53–55).. Upregulated YY1, in turn, stimulates the expression of LINC01134 by enhancing its promoter activity, establishing a positive feedback loop that drives HCC progression. Similarly, Wang et al. (43) found that LINC01134 directly binds to the promoter of AKT1 Substrate 1 (AKT1S1), further activating the NF-κB pathway and promoting HCC metastasis. Moreover, Zheng et al. (44) reported that LINC01134 may contribute to HCC carcinogenesis, at least partially, through the miR-4784/SSRP1 axis. Additionally, Yuan et al. (45) revealed that LINC01134 promotes HCC growth and metastasis by interacting with TPR and inducing the TPR-mediated transportation of p53 from the nucleus to the cytoplasm. The inactivation of p53 signaling ultimately contributes to the progression of liver cancer. These findings collectively highlight the crucial role of LINC01134 in modulating a spectrum of biological behaviors, offering valuable insights for the development of potential therapeutic strategies against HCC.

### 2.3 The role of LINC01134 in chemo- and radiotherapy resistance of HCC

**Figure 5:**
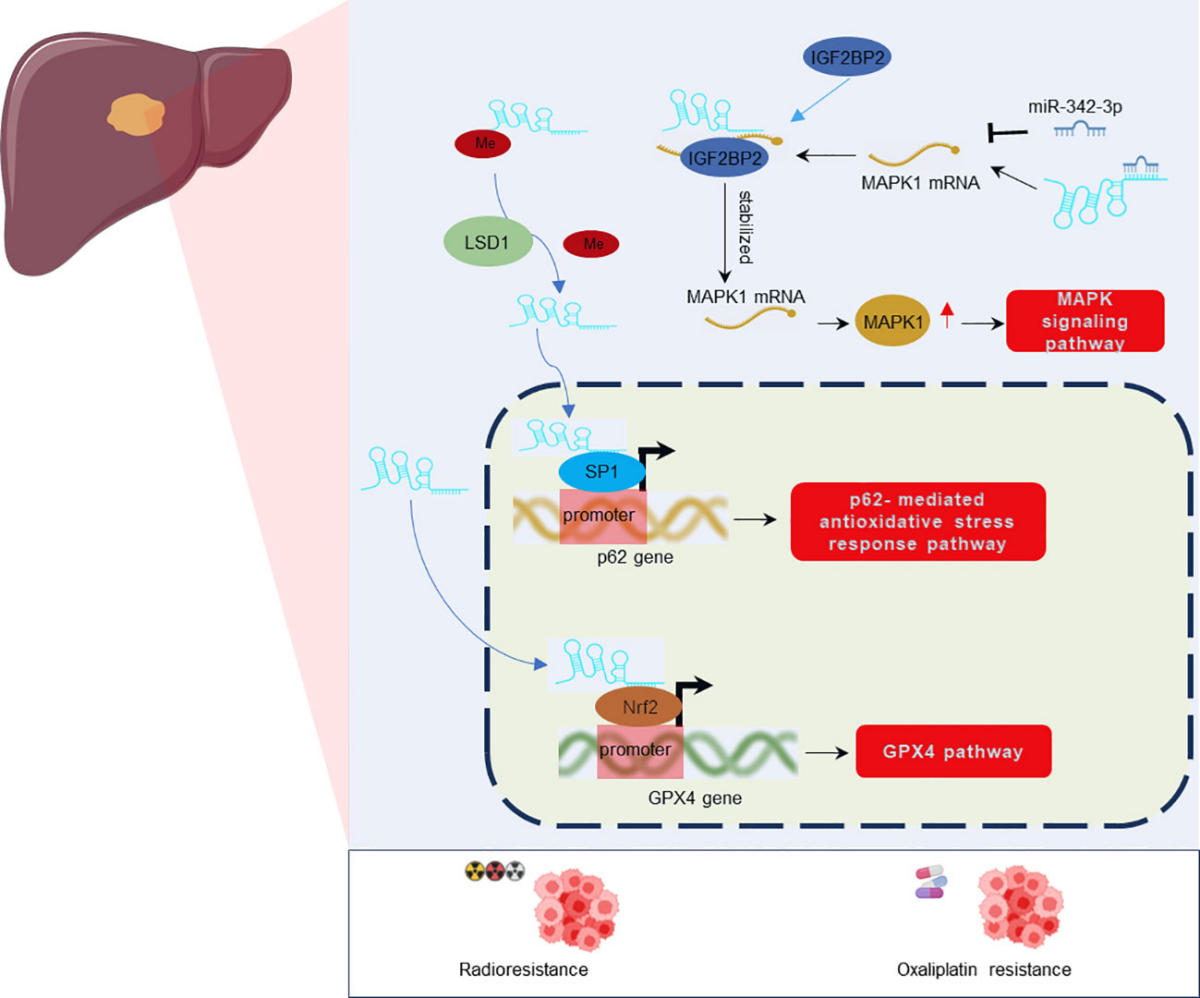
Regulatory mechanisms of LINC01134 in Oxaliplatin resistance and Radioresistance in HCC.

Resistance to chemotherapy and radiotherapy is a major obstacle in cancer treatment (56, 57). Recent investigations (49–51) have highlighted the role of LINC01134 in contributing to chemo- and radiotherapy resistance in HCC (**Figure 5**). LINC01134 has been found to modulate oxaliplatin resistance by influencing cell viability, apoptosis, and mitochondrial homeostasis through the SP1/p62 axis (49), where p62 refers to SQSTM1 or Sequestosome-1, a crucial element in this pathway. Additionally, LINC01134 functions as a negative regulator of ferroptosis and enhances resistance to oxaliplatin in hepatoma cells by activating the Glutathione Peroxidase 4 (GPX4) pathway (51). The GPX4 pathway is a critical defense mechanism against oxidative stress and lipid peroxidation in cells (58–60). Activation of this pathway is achieved through the facilitation of Nuclear Factor Erythroid 2-Related Factor 2 (Nrf2) recruitment to the GPX4 promoter (51). Furthermore, LINC01134 plays a role in the regulation of Mitogen-Activated Protein Kinase 1 (MAPK1) expression by sequestering miR-342-3p and interacting with Insulin-Like Growth Factor 2 mRNA Binding Protein 2 (IGF2BP2). This interaction subsequently activates the MAPK signaling pathway, contributing to radio-resistance in HCC cells (50). Consequently, targeting LINC01134 for downregulation using mimics could potentially enhance the sensitivity of HCC tumor cells to chemoradiotherapy, addressing the issue of treatment resistance.

### 2.4 Future clinical applications of LINC01134 in HCC

#### 2.4.1 LINC01134 as a diagnostic marker for HCC

Early detection is crucial for the successful treatment of HCC. Numerous studies have consistently reported a marked increase in LINC01134 expression in cancerous tissues of HCC patients (42–45, 50, 51), highlighting its potential as a diagnostic biomarker for timely intervention and improved patient outcomes. Notably, a significant elevation in LINC01134 expression has been observed in HCC patients who develop portal vein tumor thrombus (PVTT) (43). PVTT is a prevalent and complex complication in HCC management, linked to high recurrence rates and a poor prognosis (61, 62). These findings emphasize the vital role of LINC01134 in early prediction and prompt treatment, particularly for individuals at risk of developing PVTT.

#### 2.4.2 LINC01134 as a biomarker for HCC prognosis

The expression level of LINC01134 has been significantly correlated with HCC prognosis. Wang et al. (43) disclosed a substantial positive association between elevated LINC01134 levels and both microvascular and macrovascular invasion, critical factors in HCC staging and prognostication. Furthermore, numerous studies (42–45, 49, 51, 52, 63, 64) have reported that HCC patients with high LINC01134 expression in cancerous samples exhibit significantly poorer clinical outcomes, including reduced overall survival, diminished disease-free survival, and lower recurrence-free survival rates. In addition, several lncRNA-based prognostic models containing LINC01134, such as an 11-lncRNAs penal (52), and a 50-lncRNA-pairs (65), have been constructed and showed noticeable potential prognostic value in HCC cases. Collectively, these findings position LINC01134 as a potential prognostic biomarker for HCC.

#### 2.4.3 LINC01134 is a potential target for HCC therapy

**Figure 6:**
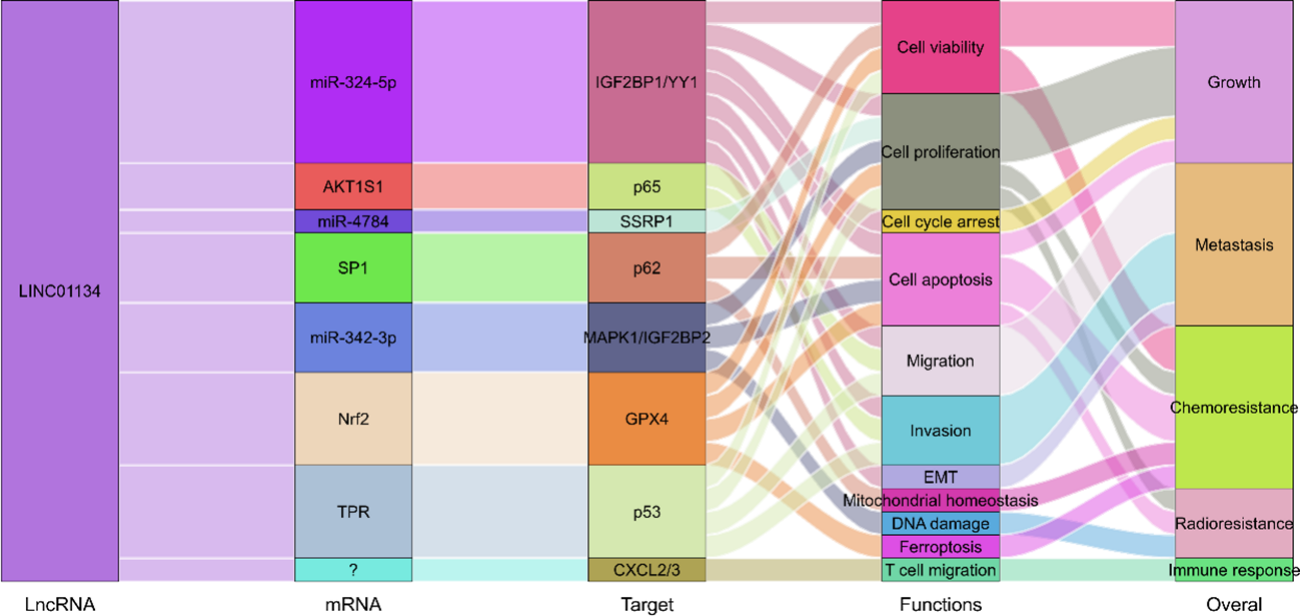
Molecular regulatory mechanisms of LINC01134 in HCC development through involvement in the regulation of multiple biological processes.

The potential of lncRNAs as therapeutic targets in cancer treatment is increasingly recognized (24, 28), and LINC01134, a newly identified lncRNA, emerges as a promising candidate for HCC therapy. Experimental evidence from multiple studies (42–45, 49–52) has elucidated the regulatory role of LINC01134 in HCC development, highlighting its influence on key molecules and genes pivotal for tumorigenesis and disease progression (**Figure 6**).

Crucially, the downregulation of LINC01134 has been shown to enhance the responsiveness of cancer cells to chemotherapy agents such as oxaliplatin (49, 51) and to mitigate radio-resistance in HCC cells (50). Moreover, a study conducted by Li et al. (52) revealed that a decrease in LINC01134 expression correlates with an upregulation of Chemokine (C-X-C motif) ligand 2 and 3, which are vital regulators in immune responses (66). They also discovered that the medium from HCC cells with reduced LINC01134 expression significantly boosts the migratory ability of Jurkat T cells, known for producing interleukin 2 (67, 68). This cytokine is closely associated with increased sensitivity to cancer drugs (69, 70) and enhanced radiation responses (71, 72).

These findings underscore the potential therapeutic implications of targeting LINC01134 in HCC treatment. Suppressing LINC01134 expression not only inhibits the malignant behaviors of HCC cells but also improves treatment response, modulates immune responses, and enhances overall therapeutic efficacy.

## 3 Perspectives

In recent times, bioinformatics has significantly advanced the discovery and annotation of lncRNAs. Mainstream technologies for sequencing and lncRNA profiling include RNA-seq and microarray, which prominently feature in repositories like The Cancer Genome Atlas (TCGA) and Gene Expression Omnibus (GEO). This advancement in bioinformatics is crucial for deeper explorations into lncRNAs. Currently, in silico methods, backed by bioinformatic analyses, stand out as powerful tools. Numerous databases are available that can be instrumental in annotating and predicting potential lncRNA functions, such as LncBook 2.0 (73) and RNAInter (74). To enrich this review, we tapped into several online lncRNA databases and high-throughput data, aiming to predict the clinical significance, functional dynamics, and pathway involvement of LINC01134 in tumorigenesis and HCC progression. These insights may provide invaluable guidance for future studies.

**Figure 7:**
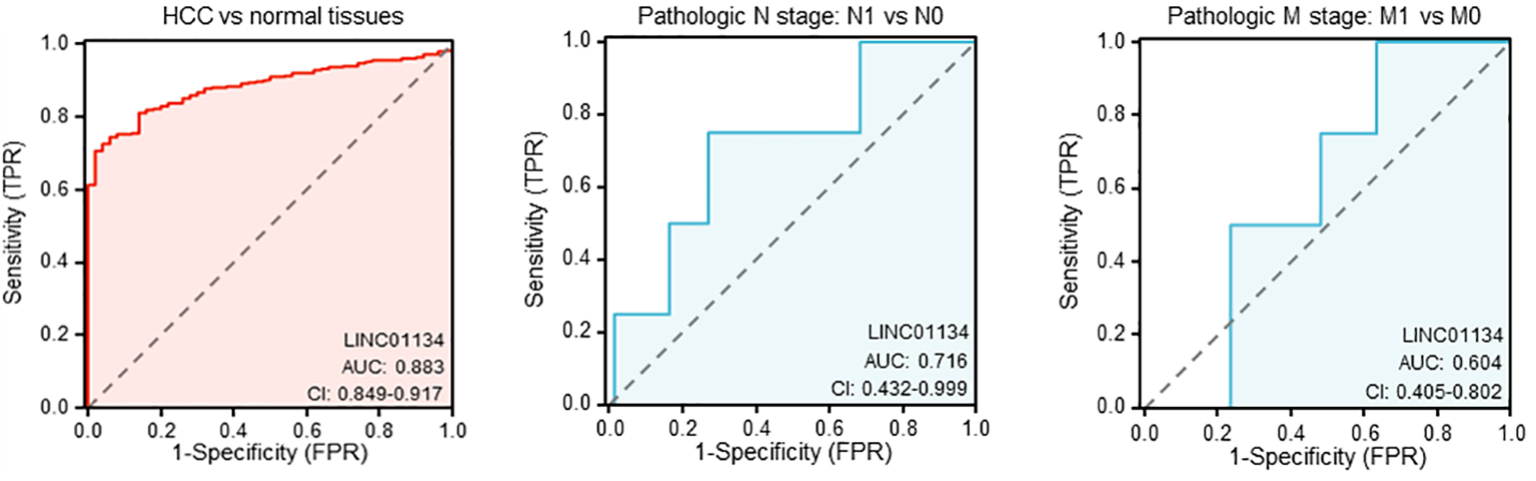
Evaluating the diagnostic utility of LINC01134 in hepatocellular carcinoma samples and their metastatic progressions.

**Figure 8:**
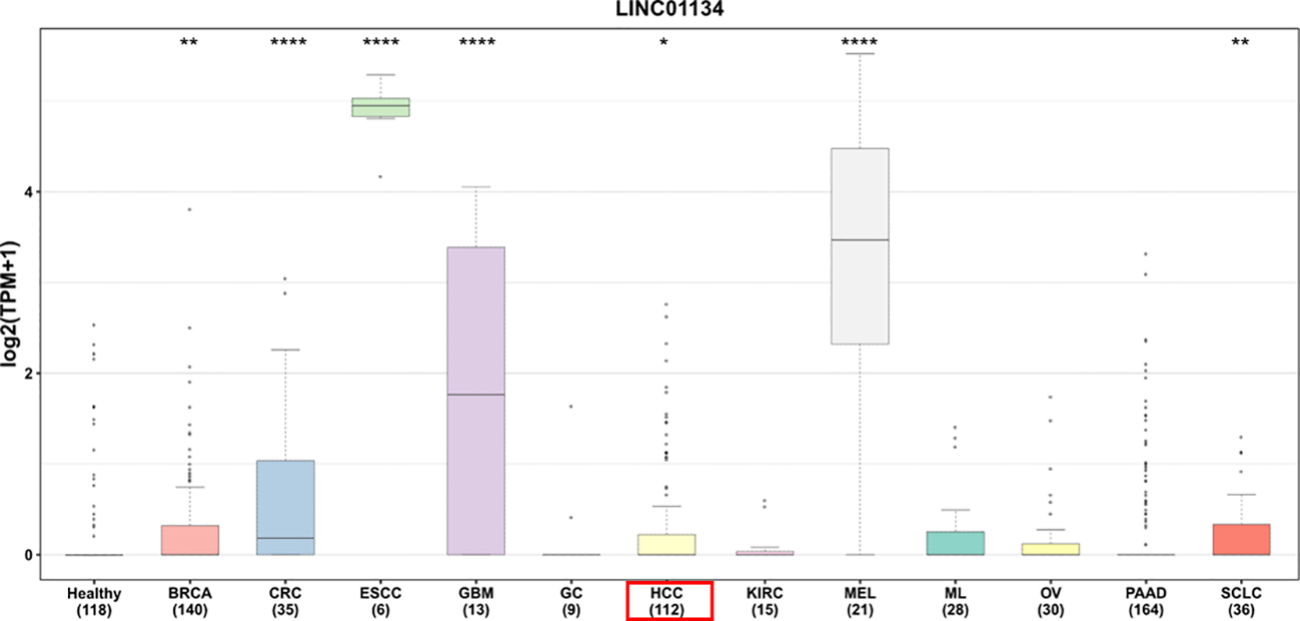
Comparative expression of LINC01134 in extracellular vesicles derived from blood across various cancer types versus healthy donors. This includes: BRCA, breast cancer; CRC, colorectal cancer; ESCC, esophageal squamous cell carcinoma; GBM, glioblastoma multiforme; GC, gastric cancer; HCC, hepatocellular carcinoma; KIRC, kidney cancer; MEL, melanoma; ML, malignant lymphoma; OV, ovarian cancer; PAAD, pancreatic adenocarcinoma; SCLC, small cell lung cancer. Significance levels are denoted by asterisks above the boxes, with * representing p < 0.05, ** for p < 0.01, and **** for p < 0.0001. Data were sourced from exoRBase (http://www.exorbase.org/).

In our investigation of LINC01134’s diagnostic potential in HCC samples, we analyzed data from the Cancer Genome Atlas Liver Hepatocellular Carcinoma (TCGA-LIHC) dataset. Our analysis revealed that LINC01134 exhibits good accuracy in distinguishing between normal and HCC tissues, as evidenced by an area under the curve (AUC) value of 0.833 (**Figure 7**). Furthermore, LINC01134 demonstrates diagnostic value in distinguishing between N0 and N1 HCC samples (AUC = 0.716) and between M0 and M1 HCC patients (AUC = 0.604) (**Figure 7**). Turning our attention to the potential of LINC01134 as a minimally invasive diagnostic tool, we initiated an analysis of extracellular vesicle lncRNA expression in blood samples. Notably, exosomes represent a subpopulation of extracellular vesicles with an average diameter of 100 nm (75). Exosomal lncRNAs are currently recognized as promising tumor diagnostic tools (76–79). Utilizing exoRBase (http://www.exorbase.org/), a repository of extracellular vesicle long RNAs derived from RNA-seq data analyses (80), we observed a significant upregulation of LINC01134 expression in the blood of individuals with various cancers, including HCC, when compared to healthy donors (**Figure 8**). These findings underscore the potential of LINC01134 as a highly promising diagnostic marker for HCC. The prospect of utilizing LINC01134 as a novel circulating oncological biomarker opens up an exciting avenue for future research.

**Figure 9:**
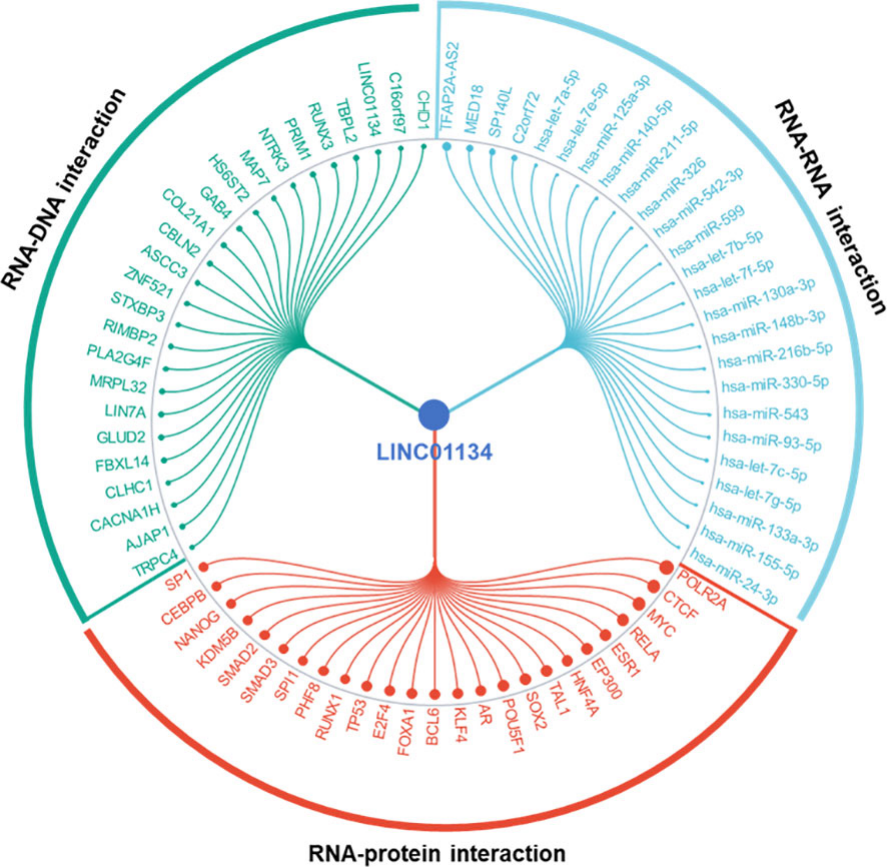
Top 25 RNA-DNA, RNA-RNA, and RNA-protein interactions associated with LINC01134. Data sourced from RNAInter (http://www.rnainter.org/).

**Figure 10:**
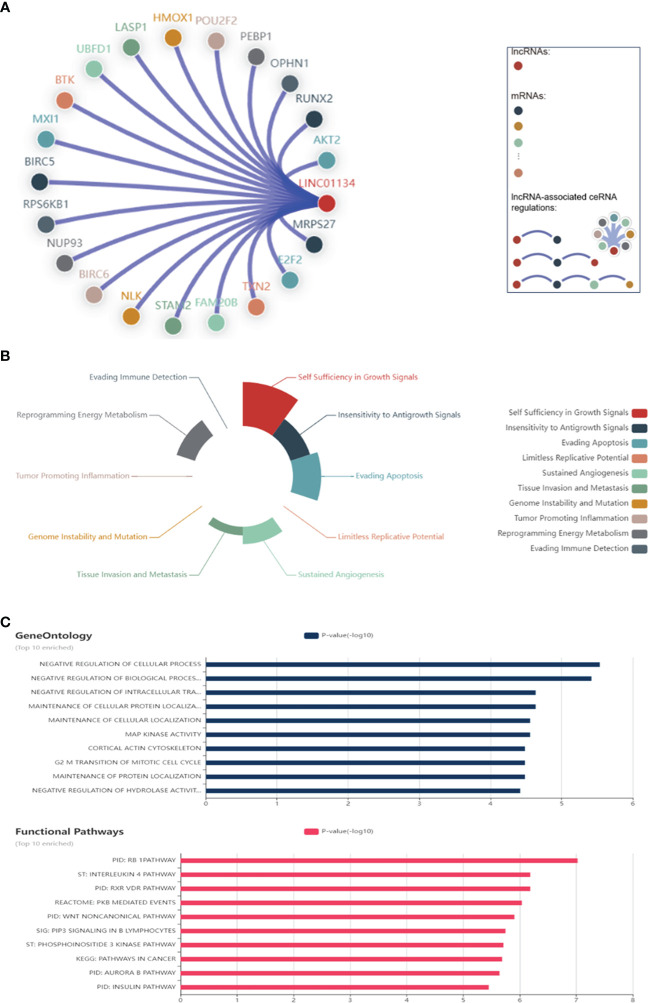
Networks Associated with LINC01134 ceRNA. **(A)** Interactions between LINC01134 and its associated ceRNA network, including corresponding mRNAs. **(B)** Principal cancer hallmarks associated with LINC01134 ceRNA, including self-sufficiency in growth signals, evasion of apoptosis, sustained angiogenesis, and tissue infiltration & metastasis. **(C)** The ten most significant functions of LINC01134-associated ceRNA, derived from gene ontology annotations and functional pathways. Data sourced from LnCeCell (http://bio-bigdata.hrbmu.edu.cn/LnCeCell/).

LncRNAs play crucial roles in modulating chromatin architecture and function, regulating transcription, and influencing RNA processing and stability (81, 82). They achieve these functions through targeting DNA, binding to RNAs, and recruiting proteins (81, 82). Here we found the top significant 25 RNA-DNA interactions, RNA-RNA interactions, and RNA-protein interactions related to LINC01134 (**Figure 9**), using RNAInter (http://www.rnainter.org/), an RNA interactome database. Accumulating evidence suggests that lncRNAs can behave like competing endogenous RNAs (ceRNAs), and compete with microRNAs to communicate with mRNAs (83–86). LncRNA-associated ceRNA regulation has been implicated in cell-fate determination and tumorigenesis (87–90). Here, we used the LnCeCell database (91) to predict lncRNA-associated ceRNA networks at single cancer cell resolution. We displayed the LINC01134-associated ceRNA-network (**Figure 10A**) and also identified LINC01134-associated ceRNA-related cancer hallmarks, such as self-sufficiency in growth signals, evading apoptosis, enduring angiogenesis, and tissue infiltration and metastasis (**Figure 10B**). Furthermore, we conducted an analysis to identify the top 10 enriched functions of LINC01134-associated ceRNA (**Figure 10C**). The findings indicate that ceRNAs associated with LINC01134 play a role in crucial tumor-related biological processes, such as the “G2-M transition of the mitotic cell cycle”, “maintenance of cellular protein localization” and “MAP kinase activity”. Furthermore, these ceRNAs are linked to several cancer-associated pathways, including the “Aurora B pathway”, “phosphoinositide 3-kinase pathway” and “Wnt non-canonical pathway”. It’s worth noting that these pathways are closely related to the onset and progression of various tumors (92–94), including HCC (95–98).

**Figure 11:**
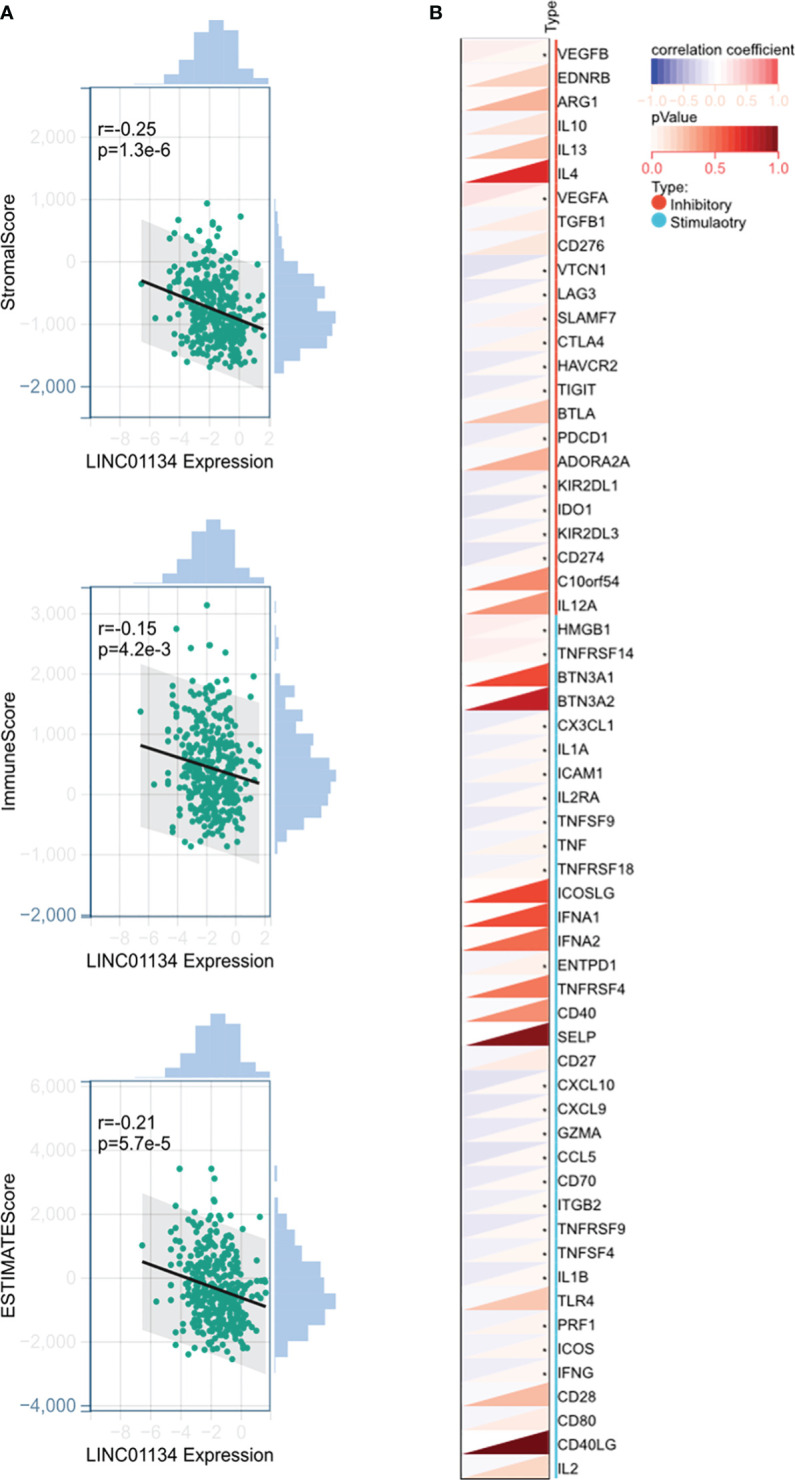
Potential Involvement of LINC01134 in HCC Immunotherapy. **(A)** Analysis shows a significant negative correlation between LINC01134 expression and immune infiltration scores, **(B)** Examination of LINC01134’s correlation with 60 genes from inhibitory and stimulatory immune checkpoint pathways. Significance levels are indicated by asterisks, with * denoting p < 0.05.

Moreover, we investigated the role of LINC01134 in immunotherapy. We obtained liver cancer datasets from UCSC (https://xenabrowser.net/) for analysis. Our findings revealed a significant negative correlation between LINC01134 gene expression and immune infiltration scores (including stromal, immune, and ESTIMATE scores) (**Figure 11A**). This suggests a potential inverse association between LINC01134 and immune infiltration, hinting at its involvement in the immune evasion mechanisms in HCC. However, more experiments and mechanistic studies are required. Additionally, we analyzed the correlation between LINC01134 and 60 key genes related to inhibitory and stimulatory immune checkpoint pathways (**Figure 11B**). The identification of LINC01134-related regulatory networks enhances our understanding of its role in tumor pathology and aids precision medicine. These predictions offer insights into potential tumor-related mechanisms, requiring further validation.

In this review, we elucidated the biological function and regulatory mechanism of LINC01134 in hepatocellular carcinoma and highlighted its potential as a diagnostic and prognosis marker, as well as a therapeutic target for HCC. However, it is imperative to emphasize that investigations into LINC01134 are at a nascent stage, and its involvement in most human tumors remains largely unexplored. Currently, LINC01134 has only been reported in the context of liver cancer, While the existing reports are limited in number, this review’s synthesis of LINC01134 research in liver cancer can serve as a valuable foundation and inspiration for future investigations in other human tumors.

Based on an in-depth analysis of previous studies, there are still some significant questions regarding the role of LINC01134 in HCC that warrant further investigation. This includes a need for further research into the biological impact of LINC01134 on other RNA-binding proteins (RBPs) (42). Furthermore, the oncogenic role of LINC01134 in HCC requires a thorough assessment through genetic knockout mouse models. Moreover, further exploration is warranted to understand the role and functions of LINC01134 in tumor stem cells of HCC (45).

Additionally, to elucidate LINC01134’s role in tumor pathogenesis, particularly in the context of drug and radiotherapy resistance, and immunotherapy impact, in-depth experimental studies of its functional and biological mechanisms are required. Furthermore, it is also necessary to investigate the expression profiles of homologous protein-coding genes associated with LINC01134. Given the significant role of endogenous siRNAs (esiRNAs) in regulating RNA silencing (99–101), future research should also focus on unraveling the interactions between esiRNAs derived from LINC01134 and their target genes. Moreover, there is a need to investigate LINC01134’s expression profiles in both tumor tissues and body fluids, aiming to assess its diagnostic potential using minimally invasive samples such as serum, which could facilitate distinguishing cancer patients from healthy individuals. Extensive research is also warranted to evaluate the prognostic value of LINC01134 across diverse populations and various pathological stages of different tumors. Undertaking these research initiatives will provide invaluable insights, paving the way for personalized cancer management in terms of diagnosis, risk stratification, and therapeutic interventions.

## 4 Conclusion

In conclusion, our study emphasizes LINC01134 as a multifaceted oncogenic lncRNA exerting a substantial impact on HCC growth, metastasis, and therapy resistance. We recognize its potential as a promising biomarker and therapeutic target in hepatoma, offering a valuable avenue for targeted interventions. However, it is imperative to recognize that further experimental investigations are necessary to comprehensively unravel the underlying roles and mechanisms of LINC01134 in hepatocarcinogenesis, progression, resistance, and immunotherapy, establishing a robust theoretical foundation. To enhance the clinical relevance of LINC01134, future clinical studies focused on this lncRNA are essential for uncovering innovative ideas and approaches in HCC diagnosis, risk stratification, and treatment.

## Author contributions

YY: Visualization, Data curation, Formal Analysis, Writing – original draft. JW: Visualization, Writing – original draft, Data curation, Formal Analysis. QG: Writing – original draft. HL: Visualization, Conceptualization, Supervision, Writing – review & editing.
